# A randomised, double-blind, placebo-controlled phase 1 study of the safety, tolerability and pharmacodynamics of volixibat in overweight and obese but otherwise healthy adults: implications for treatment of non-alcoholic steatohepatitis

**DOI:** 10.1186/s40360-018-0200-y

**Published:** 2018-03-16

**Authors:** Melissa Palmer, Lee Jennings, Debra G. Silberg, Caleb Bliss, Patrick Martin

**Affiliations:** 1 Global Development Lead Hepatology, Shire, 300 Shire Way, Lexington, MA 02421 USA; 2 Shire International GmbH, Zahlerweg 10, 6301 Zug, Switzerland

**Keywords:** Volixibat, SHP626, LUM002, Non-alcoholic steatohepatitis, Non-alcoholic fatty liver disease, Apical sodium-dependent bile acid transporter (ASBT), Cholesterol, Obesity

## Abstract

**Background:**

Accumulation of toxic free cholesterol in hepatocytes may cause hepatic inflammation and fibrosis. Volixibat inhibits bile acid reuptake via the apical sodium bile acid transporter located on the luminal surface of the ileum. The resulting increase in bile acid synthesis from cholesterol could be beneficial in patients with non-alcoholic steatohepatitis. This adaptive dose-finding study investigated the safety, tolerability, pharmacodynamics, and pharmacokinetics of volixibat.

**Methods:**

Overweight and obese adults were randomised 3:1 to double-blind volixibat or placebo, respectively, for 12 days. Volixibat was initiated at a once-daily dose of 20 mg, 40 mg or 80 mg. Based on the assessment of predefined safety events, volixibat dosing was either escalated or reduced. Other dose regimens (titrations and twice-daily dosing) were also evaluated. Assessments included safety, tolerability, stool hardness, faecal bile acid (FBA) excretion, and serum levels of 7α-hydroxy-4-cholesten-3-one (C4) and lipids.

**Results:**

All 84 randomised participants (volixibat, 63; placebo, 21) completed the study, with no serious adverse events at doses of up to 80 mg per day (maximum assessed dose). The median number of daily bowel evacuations increased from 1 (range 0–4) to 2 (0–8) during volixibat treatment, and stool was looser with volixibat than placebo. Volixibat was minimally absorbed; serum levels were rarely quantifiable at any dose or sampling time point, thereby precluding pharmacokinetic analyses. Mean daily FBA excretion was 930.61 μmol (standard deviation [SD] 468.965) with volixibat and 224.75 μmol (195.403) with placebo; effects were maximal at volixibat doses ≥20 mg/day. Mean serum C4 concentrations at day 12 were 98.767 ng/mL (standard deviation, 61.5841) with volixibat and 16.497 ng/mL (12.9150) with placebo. Total and low-density lipoprotein cholesterol levels decreased in the volixibat group, with median changes of − 0.70 mmol/L (range − 2.8 to 0.4) and − 0.6990 mmol/L (− 3.341 to 0.570), respectively.

**Conclusions:**

This study indicates that maximal inhibition of bile acid reabsorption, as assessed by FBA excretion, occurs at volixibat doses of ≥20 mg/day in obese and overweight adults, without appreciable change in gastrointestinal tolerability. These findings guided dose selection for an ongoing phase 2 study in patients with non-alcoholic steatohepatitis.

**Trial registration:**

ClinicalTrials.gov identifier: NCT02287779 (registration first received 6 November 2014).

**Electronic supplementary material:**

The online version of this article (10.1186/s40360-018-0200-y) contains supplementary material, which is available to authorized users.

## Background

Non-alcoholic steatohepatitis (NASH) is a severe, potentially progressive, fatty liver disease characterised histologically by the accumulation of excessive fat in the liver (steatosis) coupled with lobular inflammation and hepatocyte injury, with or without fibrosis [1–3]. A contributing factor to the pathogenesis of NASH is abnormal cholesterol metabolism and the accumulation of free cholesterol in the liver. Free cholesterol is directly toxic to hepatocytes, leading to inflammation and fibrosis [4].

The prevalence of NASH is difficult to establish because definitive diagnosis requires liver biopsy [5, 6]. Estimates of the population prevalence of NASH range from 2% to 5% [7]; observational studies have reported rates of approximately 5% in adults in Finland [8], and 12.2% in middle-aged adults in the US, rising to 22.2% in those with diabetes [5]. The number of people with NASH is growing at an epidemic rate, paralleling the global rise in obesity, with a prevalence of about 33% in people who are obese [6, 9]. Prospective, long-term histological follow-up studies have found that 27–43% of people with NASH develop liver fibrosis and up to 22% develop cirrhosis, depending on the study [10–14]. Progression of NASH can lead to complications such as liver failure, liver cancer, and the need for liver transplantation [15]. In recent registry studies, NASH was the second most common reason for liver transplantation in the USA in 2013 [16], and the most common reason in adults under 50 years of age in 2014 [17]. The incidence of NASH among adults awaiting liver transplantation increased by 170% from 2004 to 2013 [16].

No approved pharmacotherapies with demonstrated long-term efficacy and safety exist for NASH. Treatment guidelines for NASH recommend individualised plans to manage the metabolic comorbidities with lifestyle interventions such as weight loss, dietary changes and physical activity [18, 19]. However, guidance regarding the implementation of lifestyle interventions in the clinical setting is limited [20] and these interventions are rarely successful [19]. Untreated, NASH is associated with significant morbidity and mortality [15]. Accordingly, NASH is a disease with an unmet medical need for therapy.

Volixibat (SHP626; formerly LUM002) is a highly potent, minimally absorbed, competitive inhibitor of the apical sodium-dependent bile acid transporter (ASBT) that is being developed as a potential pharmacological treatment for NASH [21]. We hypothesise that inhibition of bile acid reuptake via ASBT will stimulate de novo synthesis of bile acids from cholesterol (including free cholesterol) in the liver and have positive metabolic, anti-inflammatory, anti-steatotic, and potentially anti-fibrotic effects in patients with NASH. Approximately 95% of bile acids that enter the gut lumen are recycled back to the gallbladder, where they are stored for subsequent release into the duodenum [22]. Inhibition of ASBT on the luminal surface of enterocytes in the terminal ileum increases faecal bile acid (FBA) excretion, with subsequent upregulation of bile acid synthesis in the liver to replenish circulating bile acids. Bile acids promote the micellisation of fats and fat-soluble vitamins to enable intestinal absorption, but also act as signalling molecules in the hepatic lipid and glucose metabolism pathways via receptors including the farnesoid X receptor (FXR) and G protein-coupled bile acid receptor 1 (GPBAR1; also known as TGR5) [23, 24]. Activation of these receptors on enteroendocrine L cells in the intestine stimulates the release of peptide hormones such as glucagon-like peptides 1 and 2 and peptide YY, which have key functions in controlling insulin release from the pancreas, modulating intestinal growth and function and regulating appetite [25–27]. Intestinal bile acid signalling also stimulates the release of fibroblast growth factors (FGFs) such as FGF19 and FGF21 [28], which regulate glucose and lipid metabolism and bile acid synthesis in the liver [29]. In the liver, bile acid signalling via FXR regulates the synthesis of bile acids, cholesterol and fatty acids, as well as controlling serum cholesterol levels [30, 31]. In addition, because bile acids are synthesized in the liver from low-density lipoprotein cholesterol (LDL-C), reduction of cholesterol levels in the liver to decrease, and possibly reverse, hepatocyte damage may be another mechanism of action for volixibat in the treatment of NASH. Indeed, it has been confirmed that serum LDL-C levels can be reduced if the recycling of bile acids via the enterohepatic circulation is inhibited [32].

In animal models, ASBT inhibitors increase bile acid excretion and promote bile acid signalling in the intestine, with resultant modulation of serum and liver bile acid concentrations, serum cholesterol levels, glucose metabolism, and hepatic fatty acid metabolism [33–35].

A previous phase 1 study [36, 37] demonstrated that volixibat, at doses of 0.5–10 mg/day for 28 days, increased FBA excretion compared with placebo, and upregulated the synthesis of new bile acids from cholesterol in the liver and serum, as indicated by dose-dependent increases in levels of serum 7α-hydroxy-4-cholesten-3-one (C4), a marker of synthesis of bile acids from cholesterol. While FBA excretion was greatest with volixibat 10 mg/day, no clear dose–response relationship was discernible at doses of 0.5–5 mg/day [36, 37]. Volixibat also reduced fasting glucose levels, suggesting improvements in glucose homeostasis in the cohort of patients with type 2 diabetes mellitus (T2DM) included in the study. Volixibat treatment commonly resulted in mild to moderate gastrointestinal adverse events (AEs), consistent with its mechanism of action and a drug with minimal systemic absorption [36, 37]. In this, and in a separate phase 1 study assessing the absorption, distribution, metabolism and excretion of [^14^C]-volixibat following a single 50 mg dose, the drug was minimally absorbed [36–38]. Serum levels of volixibat were below the lower limit of quantification in nearly all samples, so pharmacokinetic parameters for volixibat could not be calculated, even at the maximum administered dose of 50 mg/day [36–38]. The study of radiolabelled volixibat also showed that it is not metabolized and is eliminated from the body almost exclusively via faecal excretion [38].

The adaptive dose-finding phase 1 study described here reports safety, tolerability, and pharmacodynamics data of daily volixibat doses up to 80 mg (range 5-80 mg), once-daily (q.d.) or twice-daily (b.i.d.) dosing, in addition to ascending and descending dose titration regimens, in obese and overweight individuals – a population characteristic of patients with NASH. A pharmacokinetic analysis was also included to assess the systemic exposure of volixibat at doses above those investigated in previous phase 1 studies of volixibat.

## Methods

### Conduct and ethics

This phase 1 study (ClinicalTrials.gov identifier NCT02287779) was conducted between 19 January 2015 and 19 June 2015 at a single site in Knoxville, TN, USA. The study was conducted in accordance with International Conference for Harmonisation guidelines for Good Clinical Practice, the principles of the Declaration of Helsinki, and other applicable local ethical and legal requirements. The study protocol was approved by an independent institutional review board and regulatory agency before initiation. Each participant provided written informed consent before commencing any study-specific procedures.

### Participants

The study recruited generally healthy men and women aged 18–65 years who were overweight or obese (body mass index 25.0–35.0 kg/m^2^ and body weight > 63.5 kg at initial screening). ‘Generally healthy’ was defined as no evidence of any active or chronic disease following a detailed review of medical and surgical history, a complete physical examination that included monitoring of vital signs and 12-lead electrocardiography (ECG), and clinical laboratory tests (haematology, biochemistry and urinalysis). In addition, all clinical laboratory findings had to be within normal limits or be considered clinically insignificant by the study investigator. Key exclusion criteria were: a history of any haematological, hepatic, respiratory, cardiovascular, renal, neurological or psychiatric disease; gallbladder removal; and current or recurrent disease that could affect the action, absorption or disposition of the study drug, or that could affect the clinical or laboratory assessments. Full inclusion and exclusion criteria are provided in Additional file 1.

### Study design

The study comprised the following periods: screening (days − 28 to − 4), check-in (day − 3), diet stabilisation (days − 2 and − 1), treatment (days 1–12), washout (days 13 and 14), final visit (discharge, day 15) and follow-up. All participants followed an identical low-fibre (approximately 10 g/day), medium-fat (approximately 30% energy from lipids) diet that repeated every 48 h (days − 2 to 15). Eligible participants who successfully completed all the required pre-admission assessments and procedures were admitted to the clinical research centre and were randomised before dosing (day 1).

**Fig. 1:**
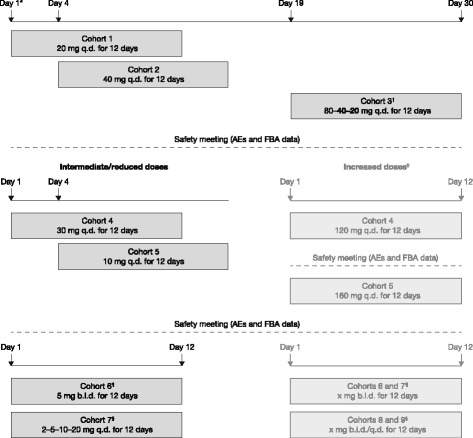
Study design. Each cohort consisted of 12 participants (volixibat, *n* = 9; placebo, *n* = 3); details are shown for the volixibat arm only. Light-grey boxes indicate dose regimen options that were not undertaken. Bold text indicates alterations to planned doses. Cohorts 4 and onwards were each initiated after reviewing results from previous cohorts.*Study days are approximate. Cohort 2 treatment was to begin at least 4 days after cohort 1 treatment; cohort 3 treatment was to start after completion of treatment in cohort 2.^†^Changed from 80 mg q.d. following review of data from cohorts 1 and 2 to a descending dose titration of 80–40–20 mg q.d. ^‡^Results from cohorts 1–3 triggered the use of intermediate and reduced doses in cohorts 4 and 5, instead of increased doses. ^¶^Treatment of an optional second b.i.d. dose cohort was not undertaken. ^§^Treatment of an optional second q.d. or b.i.d. dose titration cohort was not undertaken. AE, adverse event; b.i.d., twice daily; FBA, faecal bile acid; q.d., once daily

The study was designed to randomise participants 3:1 to receive volixibat (*n* = 9) or matching placebo (*n* = 3) for 12 days in each of up to nine planned multiple-dose cohorts (Fig. 1). Participants were randomly allocated to receive volixibat or placebo using a computer-generated randomisation schedule. Randomisation was stratified within each cohort using a block size of four. Study drugs (volixibat or placebo) were dispensed by an unblinded individual who was not involved in any other study procedure and who had minimal contact with the participants. Volixibat or matching placebo capsules were administered orally with 240 mL water, 30 min before breakfast on days 1–12. For the b.i.d. regimen, the second capsules were administered 10 h after the morning dose and 30 min before the evening meal.

This adaptive dose-finding study was designed with three initial sequential cohorts of participants who received fixed single daily doses of volixibat (cohort 1: 20 mg q.d.; cohort 2: 40 mg q.d.; cohort 3: 80 mg q.d.) (Fig. 1). Patients in cohort 2 began treatment 4 days after those in cohort 1; outcomes from cohorts 1 and 2 were evaluated before treatment was started in cohort 3 (19 days after the start of treatment in cohort 1). Modifications to the planned treatment schedule were permitted at the discretion of the investigator and the sponsor’s medical monitor, based on assessment of AEs, clinical laboratory data, ECG parameters, vital signs, and FBA excretion. The dose of volixibat was to be escalated in subsequent cohorts, provided that there were no safety or tolerability concerns and that there was a rationale for increasing the dose based on FBA excretion. Dose escalation was halted if any of three predefined safety conditions were met: (1) a serious and severe AE judged by the investigator to be related to treatment; (2) total cholesterol level < 100 mg/dL and LDL-C level ≤ 50 mg/dL on two consecutive days; or (3) clinically important AEs of moderate severity in all participants and severe AEs in at least 25% of participants in a dose cohort. If any of these conditions were met in cohorts 1–3, reduced intermediate doses were to be used for cohorts 4 and 5. Conversely, if none of the conditions were met, the dose of volixibat could be escalated to 120 mg q.d. (cohort 4) and then to 160 mg q.d. (cohort 5), with the possibility of still higher doses in cohorts 6–9. The study was also designed to assess different dose regimens (titrations or b.i.d. dosing) once the safety, tolerability and FBA excretion profiles had been established for q.d. dosing. If these profiles were similar for an initial b.i.d. dosing regimen compared with the previous q.d. regimens, then a second b.i.d dosing regimen could be examined in an additional cohort, with either an increase or decrease in b.i.d. dose. If appropriate, the study also planned to examine a titration regimen (q.d., b.i.d. or both) and determine the possibility of increasing FBA excretion while improving tolerability.

Participants in each cohort remained in the clinical research centre until discharge on day 15. A follow-up phone call was made 7 ± 2 days after the last dose of study drug to assess AEs, serious AEs and concomitant treatments.

### Objectives and outcome measures

The primary objectives of the study were to assess the safety and tolerability of multiple oral doses of volixibat administered q.d. or b.i.d. for 12 days in overweight and obese adults. The secondary objectives were: to evaluate the pharmacodynamics of volixibat using FBA and C4 concentrations and a stool assessment chart; to characterise the pharmacokinetics of multiple oral doses of volixibat administered q.d. or b.i.d. for 12 days; and to assess the safety and tolerability and characterise the pharmacokinetic profile of volixibat administered in an ascending dose titration regimen. Efficacy was not assessed in the study.

#### Safety and tolerability assessments

Safety assessments were based on AEs, physical examination, vital signs, clinical laboratory parameters, and ECG parameters. The schedule for all assessments can be found in Additional file 2: Table S1. Treatment-emergent AEs were defined as AEs that started or increased in severity on or after the first dose of study drug and up to the ninth day after treatment cessation. AEs were classified using version 17.1 of the Medical Dictionary for Regulatory Activities. Clinical laboratory assessments included serum biochemistry (at screening and on days − 3, 1, 3, 6, 9, 12, 13, and 15), haematology and urinalysis (at screening and on days − 3, 1, 6, 12, 13, and 15), serum lipid profile (at screening and on days − 3, 1, 9, 13, and 15), blood coagulation (at screening and on days − 3, 6, 13, and 15) and serum fat-soluble vitamin levels (days − 3, 13 and 15) (Additional file 2: Table S1). Blood and urine samples were taken before dosing during the treatment period.

#### Pharmacokinetic assessments

Blood samples (4 mL) for determination of plasma concentrations of volixibat were obtained at pre-dose and 0.5, 1, 1.5, 2, 3, 4, 6, 8, and 10 h after dosing on day 1; at pre-dose on days 2, 4, 7, and 10; at pre-dose and 0.5, 1, 1.5, 2, 3, 4, 6, 8, and 10 h after dosing on day 12; and on the morning of day 13. Pharmacokinetic parameters were to be determined from plasma concentration–time data for volixibat using non-compartmental analysis, including the maximum observed plasma concentration of volixibat (C_max_), the time to C_max_ (*t*_max_), and the area under the plasma concentration–time curve from time 0 to time *t* (AUC_0–*t*_), where *t* is the time of the last quantifiable plasma concentration. Plasma concentrations that were below the lower limit of quantification were reported as zero (not quantifiable).

#### Pharmacodynamic assessments

FBA and serum C4 were measured at Envigo Laboratories, Princeton Research Center, NJ, USA, using standard validated clinical laboratory tests. Blood samples (2 mL) for determination of serum concentrations of C4 were obtained on the morning of day − 1, pre-dose and 5 and 13 h after dosing on day 1, pre-dose on days 6 and 12, and on the morning of day 13.

Stool samples for determination of total FBA excretion were collected at 48-h intervals from 48 h before dosing on day 1 until day 14. Bowel movement frequency was recorded and stool hardness was assessed after each evacuation using the Bristol Stool Chart (type 1 = hardest stool, type 7 = softest stool) [39].

### Data analysis

The planned size of each cohort in this study (*n* = 12) was not based on statistical power calculations. Safety analyses were performed using the safety analysis set, defined as all randomised participants who had received at least one dose of study drug. The pharmacodynamic analysis set was defined as all participants in the safety analysis set with primary pharmacodynamic data that were considered sufficient and interpretable. The pharmacokinetic analysis set included all participants in the safety analysis set with primary pharmacokinetic data that were considered sufficient and interpretable. Statistical analyses were performed using Statistical Analysis System version 9.1.3 or higher (SAS Institute, Inc., Cary, NC, USA). Continuous variables were summarised using the following descriptive statistics: number of participants, mean, median, standard deviation (SD), minimum and maximum. Categorical and count variables were summarised using the number of participants and the percentage of participants in each category.

## Results

### Treatment cohorts

Each of the seven treatment cohorts in the study consisted of nine participants randomised to receive volixibat (volixibat group) and three randomised to receive placebo (placebo group) for 12 days (Fig. 1). Participants in the volixibat group in cohorts 1 and 2 received 20 mg q.d. and 40 mg q.d., respectively, for 12 days, as planned. Participants in the volixibat group in cohort 3 received the planned dose of 80 mg q.d. on day 1 only. The pre-specified safety condition of total cholesterol < 100 mg/dL and LDL-C ≤ 50 mg/dL on two consecutive days was met in one participant in cohort 2, triggering the use of intermediate doses below 40 mg q.d. in subsequent cohorts (this occurrence was not classified as an AE). Furthermore, participants in the volixibat group in cohort 3 received a descending q.d. dose regimen (80 mg on day 1, 40 mg on day 2, 20 mg on days 3–12) instead of the planned 80 mg q.d. Participants in the volixibat group in cohorts 4, 5, and 6 received intermediate doses (30 mg q.d., 10 mg q.d., and 5 mg b.i.d., respectively). A maximal effect based on safety, tolerability and FBA excretion was observed in cohort 2 (20 mg q.d.). Participants in the volixibat group in cohort 7 received an ascending q.d. titration regimen to the dose of maximal effect (2 mg on days 1–3, 5 mg on days 4–6, 10 mg on days 7–9, 20 mg on days 10–12).

### Participants and baseline characteristics

In total, 84 adults were randomised to receive volixibat (*n* = 63) or placebo (*n* = 21) and all participants completed the study. All randomised participants received at least one dose of study drug and were included in the safety analysis set. The pharmacodynamic analysis set consisted of 81 participants (placebo, *n* = 20; volixibat, *n* = 61); the remaining three (placebo, *n* = 1; volixibat 5 mg b.i.d., *n* = 1; volixibat 40 mg q.d., *n* = 1) were excluded from the pharmacodynamic analyses because they did not have faecal evacuations when pharmacodynamic outcomes were to be assessed. The pharmacokinetic analysis set was null because sufficient and interpretable primary pharmacokinetic data were not available for any participant.

Demographic and baseline characteristics were generally similar across the treatment groups (Table 1). At study entry, participants had a mean (± SD) age of 40.1 ± 10.93 years and a mean body mass index of 29.44 ± 2.210 kg/m^2^ and a mean weight of 90.51 ± 9.976 kg, and 78/84 (92.9%) were men.

**Table 1:** Demographic and baseline characteristics

Parameter	Placebo (*n* = 21)	Volixibat							
		5 mg b.i.d. (*n* = 9)	10 mg = q.d. (*n* = 9)	20 mg q.d. (*n* = 9)	2–5–10–20 mg q.d. (*n* = 9)	30 mg q.d. (*n* = 9)	40 mg q.d. (*n* = 9)	80–40–20 mg q.d. (*n* = 9)	Total (*n* = 63)
Age, years	41.5 (9.47)	37.1 (15.40)	46.2 (7.61)	33.3 (9.82)	46.2 (7.82)	44.6 (10.70)	36.6 (8.92)	33.2 (11.34)	39.6 (11.41)
Men, n (%)	18 (85.7)	9 (100)	7 (77.8)	9 (100)	9 (100)	8 (88.9)	9 (100)	9 (100)	60 (95.2)
BMI, kg/m^2^	29.65 (1.426)	27.81 (1.614)	29.53 (2.087)	29.49 (2.587)	29.67 (3.399)	30.96 (1.368)	28.91 (1.898)	29.27 (2.971)	29.38 (2.422)
Race, n (%)									
White	8 (38.1)	4 (44.4)	4 (44.4)	4 (44.4)	7 (77.8)	3 (33.3)	9 (100)	4 (44.4)	35 (55.6)
Black or African American	12 (57.1)	5 (55.6)	5 (55.6)	5 (55.6)	2 (22.2)	6 (66.7)	0	5 (55.6)	28 (44.4)
American Indian or Alaska native	1 (4.8)	0	0	0	0	0	0	0	0

Values are mean (standard deviation) unless otherwise stated. Data are from the safety analysis set

b.i.d., twice daily; BMI, body mass index; q.d., once daily

### Pharmacokinetic analyses

Sufficient and interpretable primary pharmacokinetic data were not available because serum levels of volixibat were rarely above the lower limit of quantification (0.0500 ng/mL) at any of the sampling time points.

### Pharmacodynamic analyses

#### FBA excretion

**Fig. 2:**
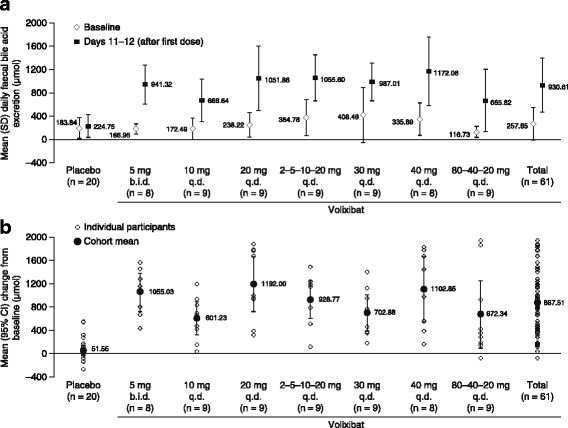
Mean daily faecal bile acid excretion (**a**) at baseline and days 11–12, and (**b**) change from baseline to days 11–12. Baseline was days − 2 and − 1 for this data set. Data are from the pharmacodynamic analysis set. b.i.d., twice daily; CI, confidence interval; q.d., once daily; SD, standard deviation

During treatment (days 1–12), the mean daily total FBA excretion was approximately fourfold greater in participants receiving volixibat than in those receiving placebo in all cohorts combined (Fig. 2a). The mean (± SD) FBA excretion after the first dose of study drug was 930.61 ± 468.965 μmol in the volixibat group, compared with 224.75 ± 195.403 μmol in the placebo group. Mean increases in FBA excretion from baseline (days − 1 and − 2) to days 11–12 in the volixibat group exceeded 600 μmol in all cohorts, while a small mean (± SD) increase from baseline (51.55 ± 180.371 μmol) was observed in the placebo group (Fig. 2b). Within the q.d. dosing cohorts, the greatest increases in FBA excretion from baseline to days 11–12 occurred at volixibat doses of 20 mg or higher. With b.i.d. dosing at 5 mg, FBA excretion was comparable to the maximal effect obtained at 20 mg q.d. or higher, and about one-third greater than that obtained at 10 mg q.d.

#### Serum C4 concentrations

**Fig. 3:**
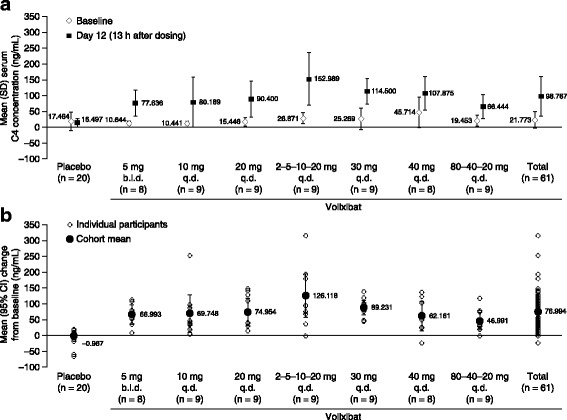
Absolute serum 7α-hydroxy-4-cholesten-3-one (C4) concentration at (**a**) baseline and 13 h after dosing on day 12, and (**b**) change from baseline to 13 h after dosing on day 12. Baseline was the last observation before the first dose of study drug. Data are from the pharmacodynamic analysis set. b.i.d., twice daily; CI, confidence interval; q.d., once daily; SD, standard deviation

On day 12, 13 h after morning dosing, mean (± SD) serum C4 concentrations were approximately sixfold higher in participants receiving volixibat (98.767 ± 61.5841 ng/mL) than in those receiving placebo (16.497 ± 12.9150 ng/mL) (Fig. 3a). In the volixibat treatment group, the highest mean (± SD) serum C4 concentration on day 12 (13 h post dose) occurred in the 2–5–10–20 mg q.d. cohort (152.989 ± 81.6364 ng/mL). Mean serum C4 concentrations in the volixibat group increased by at least 46.9 ng/mL from baseline to day 12 (13 h post dose) in all cohorts, compared with a mean (± SD) decrease of 0.967 (± 22.3963) ng/mL in the placebo group (Fig. 3b).

#### Bowel movements and stool hardness

**Fig. 4:**
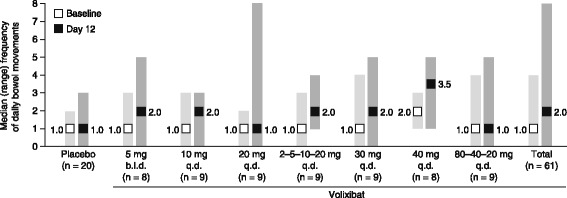
Daily frequency of bowel movements. Baseline was day − 1 for this data set. Squares represent medians; shaded bars represent ranges. Data are from the pharmacodynamic analysis set. b.i.d., twice daily; q.d., once daily

Before treatment (day − 1), the daily frequency of bowel movements and stool hardness were similar in the volixibat and placebo groups (Fig. 4). The frequency of bowel movements in all participants receiving volixibat increased from a median of 1 per day (range 0–4) at baseline (day − 1) to a median of 2 (range, 0–8) on day 12 (the last day of treatment). The increase in bowel movement frequency was not dose dependent. No meaningful changes in bowel movement frequency were observed in the placebo group during the treatment period.

The proportion of participants with stool samples rated as type 6 or 7 on the Bristol Stool Chart was numerically higher in the volixibat groups than in the placebo group during treatment (Additional file 3: Figure S1). In the volixibat group, this ranged from 41/61 participants (67.2%) on day 1 to 56/61 (91.8%) on day 11. In the placebo group, this ranged from 1/20 participants (5.0%) to 4/20 (20.0%) on days 5–10. During washout, bowel movement frequencies and stool hardness returned to pre-treatment values in both the placebo and volixibat groups.

### Clinical laboratory parameters

#### Blood lipids

The median reduction in serum total cholesterol levels from baseline to the final on-treatment assessment in the volixibat group (− 0.70 mmol/L; range − 2.8 to 0.4) was numerically greater than in the placebo group (− 0.20 mmol/L; − 1.0 to 1.7) (Table 2). Median baseline total cholesterol levels were 5.10 mmol/L (range 3.0 to 7.9) in the volixibat group and 5.50 mmol/L (3.7 to 7.5) in the placebo group. Median LDL-C levels decreased in the volixibat group during treatment (− 0.6990 mmol/L; range − 3.341 to 0.570), compared with a small increase in the placebo group (0.0260 mmol/L; − 1.088 to 1.838). Median baseline LDL-C levels were 3.0560 (range 1.321 to 5.594) in the volixibat group and 3.3930 (1.554 to 5.672) in the placebo group. No clinically meaningful changes in levels of triglycerides, high-density lipoprotein cholesterol or very low-density lipoprotein cholesterol were observed.

**Table 2:** Changes in blood lipid parameters from baseline to final on-treatment assessment

Parameter, mmol/L	Placebo (*n* = 21)	Volixibat							
		5 mg b.i.d. (*n* = 9)	10 mg q.d. (*n* = 9)	20 mg q.d. (*n* = 9)	2–5–10–20 mg q.d. (*n* = 9)	30 mg q.d. (*n* = 9)	40 mg q.d. (*n* = 9)	80–40–20 mg q.d. (*n* = 9)	Total (*n* = 63)
Total cholesterol	−0.20 (− 1.0, 1.7)	−0.90 (− 2.0, 0.3)	−0.40 (− 2.8, 0.4)	−0.70 (− 2.0, − 0.1)	−0.10 (− 0.8, 0.2)	−0.60 (− 1.4, 0.3)	−1.30 (− 1.8, − 0.1)	−1.00 (− 1.5, − 0.6)	−0.70 (− 2.8, 0.4)
LDL cholesterol	0.0260 (− 1.088, 1.838)	−0.9190 (− 1.502, − 0.233)	−0.2975 (− 2.719, 0.570)	−0.7520 (− 1.761, − 0.130)	−0.4925 (− 0.699, 0.156)	−0.6990 (− 1.451, 0.103)	−1.2690 (− 3.341, − 0.337)	−0.8030 (− 1.476, − 0.518)	−0.6990 (− 3.341, 0.570)
HDL cholesterol	− 0.030 (− 0.49, 0.73)	−0.050 (− 0.38, 0.23)	−0.080 (− 0.85, 0.18)	0.000 (− 0.26, 0.18)	0.120 (− 0.03, 0.33)	0.130 (0.00, 0.23)	0.030 (− 0.21, 0.33)	−0.050 (− 0.16, 0.16)	0.000 (− 0.85, 0.33)
Triglycerides	0.000 (− 2.49, 1.00)	0.190 (− 0.11, 3.05)	0.210 (− 0.50, 2.08)	−0.130 (− 0.68, 1.45)	−0.120 (− 0.31, 1.20)	0.060 (− 0.10, 0.32)	−0.010 (− 0.76, 1.24)	−0.200 (− 0.45, 0.05)	−0.010 (− 0.76, 3.05)
VLDL cholesterol	0.0 (−1, 1)	0.0 (0, 1)	0.0 (−1, 1)	0.0 (0, 1)	0.0 (−1, 0)	0.0 (0, 1)	0.0 (−1, 2)	0.0 (− 1, 0)	0.0 (− 1, 2)

Values are median (range). Baseline was the last observation before the first dose of study drug. Data are from the safety analysis set

b.i.d., twice daily; HDL, high-density lipoprotein; LDL, low-density lipoprotein; q.d., once daily; VLDL, very low-density lipoprotein

#### Blood glucose

No clinically meaningful changes in serum glucose levels were found during the study. At day 13, the median change in glucose concentration was − 0.20 mmol/L (range − 0.9 to 0.7) in the volixibat group and 0.00 mmol/L (− 1.5 to 0.7) in the placebo group, from median baseline concentrations of 5.20 mmol/L (3.9 to 5.9) and 5.30 mmol/L (4.2 to 7.3), respectively.

#### Fat-soluble vitamins

Clinically meaningful changes in the serum levels of vitamins A, D, E and K (as measured by prothrombin international normalised ratio [PT/INR]) were not seen during the study. Median changes in vitamin A levels at day 13 in the volixibat and placebo groups were 6.0 μg/dL (range − 19 to 31) and 4.0 μg/dL (− 22 to 18), respectively, from median baseline levels of 52.0 μg/dL (29 to 86) and 51.0 μg/dL (31 to 80), respectively. Median changes in vitamin D levels at day 13 in the volixibat and placebo groups were − 0.90 ng/mL (range − 7.4 to 8.8) and 0.50 ng/mL (− 7.3 to 6.2), respectively, from median baseline levels of 19.40 ng/mL (4.5 to 39.4) and 20.30 ng/mL (9.1 to 34.7), respectively. Median changes in vitamin E levels at day 13 in the volixibat and placebo groups were 1.00 mg/L (range − 9.6 to 8.9) and 0.90 mg/L (− 3.4 to 5.0), respectively, from median baseline levels of 9.80 mg/L (5.4 to 19.9) and 9.80 mg/L (5.7 to 16.3), respectively. Vitamin K as measured by PT/INR was unchanged at day 13 in the volixibat and placebo groups (median change of 0.00 in both groups [overall range − 0.3 to 0.8]), from a median baseline value of 1.00 (0.9 to 1.2) in both groups.

#### Alanine aminotransferase

The only clinically meaningful changes in serum biochemistry, haematology or urinalysis parameters were in serum levels of alanine aminotransferase (ALT). In the volixibat group, the median ALT level at the final on-treatment assessment had increased by 4.0 U/L (range − 44 to 78) from baseline, while a decrease was observed in the placebo group (− 2.0 U/L; − 19 to 25). During treatment, four participants had ALT levels that exceeded twice the upper limit of normal (normal range 0–55 U/L): ALT elevations started on day 3 in one participant, on day 6 in another participant, and after treatment discontinuation (on day 13) in the other two. One participant had concurrent ALT and γ glutamyl-transferase (GGT) elevations that met potentially clinically important (PCI) criteria during the treatment period, and one had concurrent ALT, aspartate aminotransferase and GGT elevations that met PCI criteria after treatment discontinuation (AST, days 14–15; GGT, days 13–18). None of these four participants with ALT elevations had elevations in bilirubin or prothrombin international normalised ratio. Furthermore, no clear dose–response relationship was evident, and ALT elevations had returned to normal or were returning to normal at the last evaluation.

### AEs

Overall, AEs were reported for the majority of participants (placebo, 14/21 [66.7%]; volixibat, 63/63 [100%]). No serious AEs or deaths were reported, and no participant discontinued treatment because of an AE. With the exception of one severe case of upper abdominal pain in a participant receiving volixibat 20 mg q.d., all AEs were mild or moderate in severity. The most commonly reported class of AEs was gastrointestinal, and the single most common AE was diarrhoea, reported for all 63 participants who received volixibat and for 11/21 participants (52.4%) who received placebo (Table 3). Diarrhoea AEs included events described as loose stools (136/182 events in the volixibat group and all 27 events in the placebo group) and events described as diarrhoea (46/182 events in the volixibat group). The majority of participants who received volixibat (39/63) experienced diarrhoea AEs from the first day of treatment. Almost all the AEs that were considered by the investigator to be related to the study drug were gastrointestinal (volixibat, 210/219 events; placebo, 28/34 events). There was no apparent relationship between the incidence of gastrointestinal AEs and volixibat dose. No changes in clinical laboratory parameters, including serum lipid profile, were reported as AEs except for one mild AE of increased hepatic enzyme (ALT) in one participant.

**Table 3:** Summary of treatment-emergent AEs

AEs, no. of events, *n* (%)	Placebo (*n* = 21)	Volixibat							
		5 mg b.i.d. (*n* = 9)	10 mg q.d. (*n* = 9)	20 mg q.d. (*n* = 9)	2–5–10–20 mg q.d. (*n* = 9)	30 mg q.d. (*n* = 9)	40 mg q.d. (*n* = 9)	80–40–20 mg q.d. (*n* = 9)	Total (*n* = 63)
Any AE	36, 14 (66.7)	44, 9 (100)	35, 9 (100)	46, 9 (100)	30, 9 (100)	21, 9 (100)	33, 9 (100)	22, 9 (100)	231, 63 (100)
AEs related to study drug	34, 12 (57.1)	39, 9 (100)	32, 9 (100)	45, 9 (100)	30, 9 (100)	18, 9 (100)	33, 9 (100)	22, 9 (100)	219, 63 (100)
AEs occurring in > 1 participant overall									
Gastrointestinal disorders									
Diarrhoea^a^	27, 11 (52.4)	33, 9 (100)	27, 9 (100)	32, 9 (100)	26, 9 (100)	16, 9 (100)	26, 9 (100)	22, 9 (100)	182, 63 (100)
Anorectal discomfort	0	1, 1 (11.1)	0	0	0	2, 2 (22.2)	1, 1 (11.1)	0	4, 4 (6.3)
Nausea	0	1, 1 (11.1)	1, 1 (11.1)	2, 1 (11.1)	1, 1 (11.1)	0	0	0	5, 4 (6.3)
Abdominal pain	1, 1 (4.8)	0	1, 1 (11.1)	2, 2 (22.2)	0	0	0	0	3, 3 (4.8)
Abdominal pain, upper	0	0	0	3, 3 (33.3)	0	0	0	0	3, 3 (4.8)
Gastrointestinal sounds, abnormal	0	0	1, 1 (11.1)	0	1, 1 (11.1)	0	1, 1 (11.1)	0	3, 3 (4.8)
Vomiting	0	1, 1 (11.1)	0	2, 1 (11.1)	1, 1 (11.1)	0	0	0	4, 3 (4.8)
Defaecation urgency	0	0	0	2, 2 (22.2)	0	0	0	0	2, 2 (3.2)
Nervous system disorders									
Headache	2, 2 (9.5)	1, 1 (11.1)	0	1, 1 (11.1)	1, 1 (11.1)	0	3, 2 (22.2)	0	6, 5 (7.9)
General disorders and administrative site conditions									
Application site irritation	1, 1 (4.8)	0	1, 1 (11.1)	1, 1 (11.1)	0	0	0	0	2, 2 (3.2)
Pyrexia	0	2, 2 (22.2)	0	0	0	0	0	0	2, 2 (3.2)

Values are the number of events, followed by the number and percentage of participants experiencing the event [m, n (%)]. Data are from the safety analysis set

^a^Includes events described as ‘loose stools’ or ‘diarrhoea’

AE, adverse event; b.i.d., twice daily; q.d., once daily

### Vital signs

The only clinically meaningful changes in vital signs or ECG parameters related to participants’ weight. A reduction in median weight was observed at day 15 in both treatment groups. In the volixibat group, reductions in median weight were observed in all cohorts except for the 2–5–10–20 mg q.d. cohort. Compared with a change of − 1.20 kg (range, − 7.20 to 1.9) in the placebo group, reductions were numerically greater in the cohorts receiving volixibat 20 mg q.d. (− 3.60 kg [range − 6.6 to − 0.4]), 80–40–20 mg q.d. (− 3.5 kg [− 6.9 to − 0.6]), 40 mg q.d. (− 2.90 kg [− 5.7 to − 0.7]), and 30 mg q.d. (− 2.30 kg [− 4.9 to 0.3]). At day 15, the proportion of participants with a PCI reduction in weight (≥5% from baseline) was 11/63 (17.5%) in the volixibat group and 1/21 (4.8%) in the placebo group, with the highest proportion occurring in the 20 mg q.d. cohort (4/9; 44.4%) and the lowest in the 2–5–10–20 mg q.d. cohort (0/8; 0%). No clear relationship between dose and degree of weight reduction was evident.

## Discussion

This double-blind, randomised, placebo-controlled, multiple-dose, phase 1 study characterised the safety, tolerability, and pharmacodynamics of oral volixibat in overweight and obese adults. In agreement with a previous phase 1 study of volixibat [36, 37], inhibition of ASBT was generally found to be well tolerated and resulted in increased FBA excretion and bile acid synthesis (indicated by increased serum C4 levels). Furthermore, reductions in total cholesterol and LDL-C levels were numerically greater in the volixibat group than in the placebo group but there was no evidence of an effect on glucose levels. In a previous phase 1 study of volixibat in adults with T2DM [36, 37], fasting glucose levels were nominally significantly lower with volixibat than with placebo, with a trend towards improved insulin sensitivity, but there was no evidence for an effect on cholesterol levels [36, 37]. Taken together, the decreased cholesterol levels in normoglycaemic patients with obesity in the present study and the decreased fasting glucose levels in normolipidaemic patients with T2DM in the previous phase 1 study suggest that volixibat may have both anti-dyslipidaemic and anti-dysglycaemic effects in patients with NASH, who typically have obesity, T2DM, or both [40].

Consistent with previous studies of volixibat at doses up to 50 mg [36–38], in the present study volixibat at doses up to 80 mg was minimally absorbed. Due to the lack of sufficient and interpretable primary pharmacokinetic data, pharmacokinetic parameters could not be calculated for volixibat. The minimal absorption of volixibat is most likely due its benzothiepine-based structure including a negatively charged sulfonate moiety [41], which is thought to prevent an interaction with the intestinal cell membrane. The low bioavailability of volixibat is expected considering its mechanism of action as a local inhibitor in the intestinal lumen. Importantly, high systemic exposure of volixibat is neither necessary nor desirable in the treatment of diseases such as NASH, because the local inhibition of bile acid reabsorption in the small intestine results in a series of systemic downstream reactions involved in cholesterol and glucose metabolic processes. Furthermore, the low bioavailability of volixibat reduces the potential for interactions with other pharmacotherapies that are likely to be administered for associated comorbidities in patients with NASH.

The most commonly reported AEs were gastrointestinal, mainly diarrhoea or loose stools. Elevation of the concentration of bile acids in the colon increases mucus secretion, stimulates colonic contractions and reduces colonic transit time [42]. Only one participant receiving volixibat had a severe AE of abdominal pain (at a dose of 20 mg q.d.). The observed AE outcomes were consistent with those seen in a previous phase 1 study, in which diarrhoea was the most frequently reported AE in participants receiving volixibat 10 mg q.d. for 28 days [36, 37]. Neither ascending nor descending volixibat titration (2–5–10–20 mg q.d. or 80–40–20 mg q.d.) provided any clinically significant improvement in tolerability.

Daily oral volixibat for 12 days inhibited bile acid reabsorption, as indicated by increases in FBA excretion and the median frequency of bowel movements relative to baseline and placebo. An associated upregulation of bile acid synthesis from cholesterol was demonstrated by increases in serum C4 concentration relative to baseline and placebo. Furthermore, median reductions in total blood cholesterol and LDL-C levels were numerically greater in patients receiving volixibat than in those receiving placebo. In a previous phase 1 study that investigated daily volixibat doses of 0.5, 1, 5 and 10 mg for 28 days, the greatest increases in FBA excretion and serum C4 concentration were found in the volixibat 10 mg q.d. cohort [36, 37]. In the present study, there was evidence of a dose-dependent effect of volixibat on FBA excretion in the q.d. dose cohorts. The highest mean increases in FBA excretion after the first dose of volixibat occurred in the 20 mg q.d. and 40 mg q.d. cohorts, and FBA excretion in the 10 mg q.d. cohort was approximately two-thirds of the maximal effect. The present study also investigated whether b.i.d. dosing was more effective than q.d. dosing. In patients receiving volixibat 5 mg b.i.d., FBA excretion was similar to the maximum levels seen with q.d. dosing, although in practice the occurrence of diarrhoea after the second dose is likely to limit the feasibility of a b.i.d. dosing regimen.

Due to the lack of primary pharmacokinetic data, a traditional exposure-response analysis using PK/PD modelling was not possible. However, the study provides an alternative approach to the characterization of a dose-response relationship of volixibat, with response based on a marker for potential clinical efficacy (FBA excretion) and on tolerability. The results show that a volixibat dose of 20 mg q.d. resulted in near-maximal increases in FBA excretion, with no apparent relationship between volixibat dose and the incidence of gastrointestinal AEs.

A median increase in ALT level (4.0 U/L higher than baseline) was observed in the volixibat group but not in the placebo group. These mild, asymptomatic ALT elevations, which were not dose dependent, may be an expected and transient effect of ASBT inhibitors, resulting from increased hepatic cholesterol turnover, rather than an adverse effect of the drug [43, 44]. Phase 2 studies of volixibat will investigate this possibility further.

Fat-soluble vitamin deficiency was not reported in this 12-day study. Because bile acids play a crucial role in the absorption of fat-soluble vitamins, further evaluation of vitamin A, D, E and K levels is warranted in ongoing trials of longer duration.

## Conclusion

This phase 1 study of volixibat in overweight and obese adults involved a short treatment period and a small sample size, factors that may limit interpretation of the results. Nevertheless, the promising safety and tolerability profile of volixibat, combined with the observed effects on bile acids and lipid metabolism, warrant further clinical development of this ASBT inhibitor. Based on the results of this trial and other phase 1 studies of volixibat [36, 37], as well as preclinical studies of ASBT inhibition [45], a 48-week, double-blind, placebo-controlled, phase 2 study in adults with NASH (ClinicalTrials.gov Identifier: NCT02787304) has been started. The study aims to assess the effects of volixibat 5, 10 and 20 mg q.d. compared with placebo on liver histology, serum liver-related biochemistry, serum lipids and metabolic indicators (blood glucose, insulin, glycated haemoglobin), in addition to safety and tolerability.

## Additional files

Additional file 1: Inclusion and exclusion criteria. (PDF 65 kb)

Additional file 2: **Table S1.** Schedule of study assessments. (PDF 69 kb)

Additional file 3: **Figure S1.** Frequency of stool hardness during treatment (Bristol Stool Chart scores). (PDF 1081 kb)

## Abbreviations

AE: adverse event
ALT: alanine aminotransferase
ASBT: apical sodium-dependent bile acid transporter
AUC_0–*t*_: area under the plasma concentration–time curve from time 0 to time *t*
b.i.d.: twice-daily
CI: confidence interval
C_max_: maximum observed plasma concentration
ECG: electrocardiography
FGF: fibroblast growth factor
FXR: farnesoid X receptor
GGT: γ glutamyl-transferase
GPBAR1: G protein-coupled bile acid receptor 1
HDL-C: high-density lipoprotein cholesterol
LDL-C: low-density lipoprotein cholesterol
NASH: non-alcoholic steatohepatitis
PCI: potentially clinically important
PT/INR: prothrombin international normalised ratio
q.d.: once daily
SD: standard deviation
T2DM: type 2 diabetes mellitus
*t*_max_: time to maximum observed plasma concentration
VLDL-C: very low-density lipoprotein cholesterol

## References

[1] Chalasani N, Younossi Z, Lavine JE, Diehl AM, Brunt EM, Cusi K, Charlton M, Sanyal AJ (2012). The diagnosis and management of non-alcoholic fatty liver disease: practice guideline by the American Association for the Study of Liver Diseases, American College of Gastroenterology, and the American Gastroenterological Association. Hepatology.

[2] Masuoka HC, Chalasani N (2013). Nonalcoholic fatty liver disease: an emerging threat to obese and diabetic individuals. Ann N Y Acad Sci.

[3] Rinella ME (2015). Nonalcoholic fatty liver disease: a systematic review. JAMA.

[4] Musso G, Gambino R, Cassader M (2013). Cholesterol metabolism and the pathogenesis of non-alcoholic steatohepatitis. Prog Lipid Res.

[5] Williams CD, Stengel J, Asike MI, Torres DM, Shaw J, Contreras M, Landt CL, Harrison SA (2011). Prevalence of nonalcoholic fatty liver disease and nonalcoholic steatohepatitis among a largely middle-aged population utilizing ultrasound and liver biopsy: a prospective study. Gastroenterology.

[6] Zezos P, Renner EL (2014). Liver transplantation and non-alcoholic fatty liver disease. World J Gastroenterol.

[7] Bhala N, Jouness RI, Bugianesi E (2013). Epidemiology and natural history of patients with NAFLD. Curr Pharm Des.

[8] Hyysalo J, Mannisto VT, Zhou Y, Arola J, Karja V, Leivonen M, Juuti A, Jaser N, Lallukka S, Kakela P (2014). A population-based study on the prevalence of NASH using scores validated against liver histology. J Hepatol.

[9] Vernon G, Baranova A, Younossi ZM (2011). Systematic review: the epidemiology and natural history of non-alcoholic fatty liver disease and non-alcoholic steatohepatitis in adults. Aliment Pharmacol Ther.

[10] Ekstedt M, Franzen LE, Mathiesen UL, Thorelius L, Holmqvist M, Bodemar G, Kechagias S (2006). Long-term follow-up of patients with NAFLD and elevated liver enzymes. Hepatology.

[11] Wong VW, Wong GL, Choi PC, Chan AW, Li MK, Chan HY, Chim AM, Yu J, Sung JJ, Chan HL (2010). Disease progression of non-alcoholic fatty liver disease: a prospective study with paired liver biopsies at 3 years. Gut.

[12] Fassio E, Alvarez E, Dominguez N, Landeira G, Longo C (2004). Natural history of nonalcoholic steatohepatitis: a longitudinal study of repeat liver biopsies. Hepatology.

[13] Adams LA, Sanderson S, Lindor KD, Angulo P (2005). The histological course of nonalcoholic fatty liver disease: a longitudinal study of 103 patients with sequential liver biopsies. J Hepatol.

[14] McPherson S, Hardy T, Henderson E, Burt AD, Day CP, Anstee QM (2015). Evidence of NAFLD progression from steatosis to fibrosing-steatohepatitis using paired biopsies: implications for prognosis and clinical management. J Hepatol.

[15] Angulo P (2002). Nonalcoholic fatty liver disease. N Engl J Med.

[16] Wong RJ, Aguilar M, Cheung R, Perumpail RB, Harrison SA, Younossi ZM, Ahmed A (2015). Nonalcoholic steatohepatitis is the second leading etiology of liver disease among adults awaiting liver transplantation in the United States. Gastroenterology.

[17] Banini BA. Nonalcoholic steatohepatitis (NASH) has surpassed hepatitis C as the leading etiology for listing for liver transplant: implications for NASH in children and young adults. In: American College of Gastroenterology Annual Scientific Meeting (Congress Abstract 46). 2016. https://www.eventscribe.com/2016/ACG/QRcode.asp?Pres=199366 Accessed 17 Nov 2017.

[18] European Association for the Study of the Liver (2016). European Association for the Study of diabetes, European Association for the Study of obesity: EASL-EASD-EASO clinical practice guidelines for the management of non-alcoholic fatty liver disease. J Hepatol.

[19] Kassirer JP, Angell M (1998). Losing weight – an ill-fated new Year's resolution. N Engl J Med.

[20] Hallsworth K, Avery L, Trenell MI (2016). Targeting lifestyle behavior change in adults with NAFLD during a 20-min consultation: summary of the dietary and exercise literature. Curr Gastroenterol Rep.

[21] Keller B, Dorenbaum A, Wynne D, Gedulin B, Setchell K, Olek E, Levin N, Kennedy C. Effect of apical sodium-dependent bile acid transporter (ASBT) inhibition on serum and fecal bile acids in healthy volunteers (congress abstract 55). Falk Symposium. 2014;194

[22] Shneider BL (2001). Intestinal bile acid transport: biology, physiology, and pathophysiology. J Pediatr Gastroenterol Nutr.

[23] Halilbasic E, Claudel T, Trauner M (2013). Bile acid transporters and regulatory nuclear receptors in the liver and beyond. J Hepatol.

[24] Hylemon PB, Zhou H, Pandak WM, Ren S, Gil G, Dent P (2009). Bile acids as regulatory molecules. J Lipid Res.

[25] Drucker DJ, Nauck MA (2006). The incretin system: glucagon-like peptide-1 receptor agonists and dipeptidyl peptidase-4 inhibitors in type 2 diabetes. Lancet.

[26] Dunning BE, Foley JE, Ahren B (2005). Alpha cell function in health and disease: influence of glucagon-like peptide-1. Diabetologia.

[27] Gutzwiller JP, Goke B, Drewe J, Hildebrand P, Ketterer S, Handschin D, Winterhalder R, Conen D, Beglinger C (1999). Glucagon-like peptide-1: a potent regulator of food intake in humans. Gut.

[28] Cyphert HA, Ge X, Kohan AB, Salati LM, Zhang Y, Hillgartner FB (2012). Activation of the farnesoid X receptor induces hepatic expression and secretion of fibroblast growth factor 21. J Biol Chem.

[29] Pournaras DJ, Glicksman C, Vincent RP, Kuganolipava S, Alaghband-Zadeh J, Mahon D, Bekker JH, Ghatei MA, Bloom SR, Walters JR (2012). The role of bile after roux-en-Y gastric bypass in promoting weight loss and improving glycaemic control. Endocrinology.

[30] Lambert G, Amar MJ, Guo G, Brewer HB, Gonzalez FJ, Sinal CJ (2003). The farnesoid X-receptor is an essential regulator of cholesterol homeostasis. J Biol Chem.

[31] Modica S, Gadaleta RM, Moschetta A (2010). Deciphering the nuclear bile acid receptor FXR paradigm. Nucl Recept Signal.

[32] Halilbasic E, Baghdasaryan A, Trauner M (2013). Nuclear receptors as drug targets in cholestatic liver diseases. Clin Liver Dis.

[33] Chen L, Yao X, Young A, McNulty J, Anderson D, Liu Y, Nystrom C, Croom D, Ross S, Collins J (2012). Inhibition of apical sodium-dependent bile acid transporter as a novel treatment for diabetes. Am J Physiol Endocrinol Metab.

[34] Gedulin B. Apical sodium-dependent bile transport inhibitors (ASBTi) exhibit potent antidiabetic activity in ZDF rats. 49th EASD Annual Meeting.

[35] West KL, Zern TL, Butteiger DN, Keller BT, Fernandez ML (2003). SC-435, an ileal apical sodium co-dependent bile acid transporter (ASBT) inhibitor lowers plasma cholesterol and reduces atherosclerosis in Guinea pigs. Atherosclerosis.

[36] Tiessen RG, Kennedy C, Keller B, Levin N, Acevedo L, Gedulin C, van Vliet A, Dorenbaum A, Palmer M. Randomized controlled trial: safety, tolerability, pharmacokinetics and pharmacodynamics of apical sodium-dependent bile acid transporter inhibition with volixibat in healthy adults and patients with type 2 diabetes mellitus. BMC Gasteroenterology. 2017; [ms submitted] 10.1186/s12876-017-0736-0 PMC5756385 29304731

[37] Tiessen RG, Kennedy C, Keller BT, Levin N, Acevedo L, Wynne D, Gedulin B, van Vliet A, Olek E, Dorenbaum A: LUM002 positive metabolic profile shown after administration of 10mg for 28 days in type 2 diabetes mellitus patients leading to potential treatment for patients with nonalcoholic steatohepatitis (NASH). Hepatology 2014, 60(S1):629A.

[38] Siebers N, Palmer M, Silberg DG, Jennings L, Bliss C, Martin PT. Absorption, Distribution, Metabolism, and excretion of [14C]-Volixibat in healthy men: phase 1 open-label study. Eur J Drug Metab Pharmacokinet. 2017; 10.1007/s13318-017-0429-7. [Epub ahead of print] 10.1007/s13318-017-0429-7 PMC5794849 28702877

[39] Lewis SJ, Heaton KW (1997). Stool form scale as a useful guide to intestinal transit time. Scand J Gastroenterol.

[40] Chiang DJ, Pritchard MT, Nagy LE (2011). Obesity, diabetes mellitus, and liver fibrosis. Am J Physiol Gastrointest Liver Physiol.

[41] Tremont SJ, Lee LF, Huang HC, Keller BT, Banerjee SC, Both SR, Carpenter AJ, Wang CC, Garland DJ, Huang W (2005). Discovery of potent, nonsystemic apical sodium-codependent bile acid transporter inhibitors (part 1). J Med Chem.

[42] Wilcox C, Turner J, Green J (2014). Systematic review: the management of chronic diarrhoea due to bile acid malabsorption. Aliment Pharmacol Ther.

[43] Chalasani N (2005). Statins and hepatotoxicity: focus on patients with fatty liver. Hepatology.

[44] Herzog E, Pragst I, Waelchli M, Gille A, Schenk S, Mueller-Cohrs J, Diditchenko S, Zanoni P, Cuchel M, Seubert A (2016). Reconstituted high-density lipoprotein can elevate plasma alanine aminotransferase by transient depletion of hepatic cholesterol: role of the phospholipid component. J Appl Toxicol.

[45] Rao A, Kosters A, Mells JE, Zhang W, Setchell KD, Amanso AM, Wynn GM, Xu T, Keller BT, Yin H (2016). Inhibition of ileal bile acid uptake protects against nonalcoholic fatty liver disease in high-fat diet-fed mice. Sci Transl Med.

