# Supplementary material for: A randomised, double-blind, placebo-controlled phase 1 study of the safety, tolerability and pharmacodynamics of volixibat in overweight and obese but otherwise healthy adults: implications for treatment of non-alcoholic steatohepatitis
Source: BMC Pharmacol Toxicol. 2018 Mar 16;19:10. doi: 10.1186/s40360-018-0200-y (PMC5857122; doi:10.1186/s40360-018-0200-y)
Supplement: Supplementary file 1 — Inclusion and exclusion criteria. (PDF 65 kb) [file 40360_2018_200_MOESM1_ESM.pdf]

Appendix S1: inclusion and exclusion criteria

Inclusion criteria

Each participant had to meet all of the following criteria to be eligible for the study.

1. The participant had the understanding, ability and willingness to comply fully with study procedures, study diet and restrictions.

2. The participant had the ability to provide voluntarily written, signed and dated (personally or via a legally authorised representative) informed consent as applicable to participate in the study.

3. The participant was aged 18–65 years inclusive at the time of consent. The date of signature of the informed consent was defined as the beginning of the screening period. This inclusion criterion was assessed at the first screening visit.

4. The participant was a man who agreed to comply with any applicable contraceptive requirements of the protocol or a woman who was not of childbearing potential.

5. The participant was considered to be generally healthy. Health status was defined as the absence of evidence of any active or chronic disease after a detailed medical and surgical history, a complete physical examination including monitoring of vital signs, 12-lead electrocardiography (ECG) and clinical laboratory tests (haematology, biochemistry and urinalysis).

6. The participant had a body mass index of 25.0–35.0 kg/m² (inclusive) with a body weight >63.5 kg (140 lb). This inclusion criterion was assessed only at the first screening visit.

7. All clinical laboratory parameters for the participant were within normal limits or were not found to be clinically significant by the investigator.

8. The participant had the ability to swallow study drug capsules.

Exclusion criteria

Participants who met any of the following criteria were excluded from the study.

1. The participant had a history of any haematological, hepatic, respiratory, cardiovascular, renal, neurological or psychiatric disease, gallbladder removal, or current or recurrent disease that could affect the action, absorption, or disposition of the study drug or clinical or laboratory assessments.

2. The participant had a current or relevant history of physical or psychiatric illness, any medical disorder that may have required treatment or made the participant unlikely to complete the study fully, or any condition that presented undue risk from the study drug or procedures.

3. The participant had a known or suspected intolerance or hypersensitivity to the study drugs, closely related compounds or any of the stated ingredients.

4. The participant had significant illness, as judged by the investigator, within 2 weeks of the first dose of study drug.

5. The participant had a known history of alcohol or other substance abuse within the last year.

6. The participant had donated blood or blood products (e.g. plasma or platelets) within 60 days before receiving the first dose of study drug.

7. If, within 30 days before the first dose of study drug, the participant had used a study drug (if its elimination half-life was less than 6 days, otherwise five half-lives),

had been enrolled in a clinical study (including vaccine studies) that, in the investigator’s opinion, may have impacted on this Shire-sponsored study or had had any substantial changes in eating habits (except for study-mandated changes in meals) or exercise routine, as assessed by the investigator.

8. The participant had confirmed systolic blood pressure >145 mmHg or <89 mmHg and diastolic blood pressure >95 mmHg or <49 mmHg.

9. The participant had 12-lead ECG showing a QT interval corrected by the Fridericia formula (QTcF) of >460 ms for men or >470 ms for women at screening. If the QTcF exceeded these values, the ECG was repeated twice and the average of the three QTcF values was used to determine eligibility.

10. The participant had a positive screen for drugs of abuse at screening or a positive screen for alcohol and drugs of abuse on day −3 of the treatment period.

11. The participant consumed >21 units of alcohol per week (3 per day) for men or >14 units per week (2 per day) for women.

12. The participant had had a positive human immunodeficiency virus, hepatitis B surface antigen or hepatitis C virus antibody screen.

13. The participant used tobacco in any form (e.g. smoking or chewing) or other nicotine-containing products in any form (e.g. gum, patch). Ex-users were to report that they had stopped using tobacco for at least 30 days before receiving the first dose of study drug.

14. The participant routinely consumed more than 2 units of caffeine per day or experienced caffeine withdrawal headaches.

15. The participant had previously failed screening, randomisation, participation or enrolment in this study. If participants had successfully completed a study cohort,

they may have participated further providing at least 30 days had passed since their last dose of study drug and they had successfully completed all rescreening procedures.

16. The participant was currently using any medication (including over-the-counter, herbal or homeopathic preparations), with the exception of the following: hormone replacement therapy, 3-hydroxy-3-methylglutaryl-coenzyme A reductase inhibitors (statins), histamine H2 antagonists, histamine H1 antagonists (non-drowsy formula), thyroid hormone replacement therapy, antihypertensive therapy with the exclusion of the diuretic class (hydrochlorothiazide was permitted), non-steroidal anti-inflammatory drugs (with the exception of celecoxib), multivitamins (as a single preparation; if individual vitamins were taken, vitamin E was not allowed), selective serotonin reuptake inhibitors (these were to be discussed with the medical monitor before any study drug administration) and allergy immunotherapy injections (participants were not allowed to leave the clinical research centre for these injections once they had checked in at day −3 until discharge or early termination). Current use was generally defined as use within 14 days of the first dose of study drug.

17. The participant was unable to follow the study-specific standardised diet and meal schedule or was unable to fast, as required during the study.

18. The participant had undergone a colonoscopy, barium enema or other test that required bowel cleansing within 4 weeks before the first dose of study drug.

19. The participant reported typically having fewer than three bowel movements per week or more than three per day.

20. The participant had used antibiotics within 30 days of the first dose of study drug.

21. The participant had used bile acid sequestrants within 30 days of the first dose of study drug.
