# Supplementary material for: A randomised, double-blind, placebo-controlled phase 1 study of the safety, tolerability and pharmacodynamics of volixibat in overweight and obese but otherwise healthy adults: implications for treatment of non-alcoholic steatohepatitis
Source: BMC Pharmacol Toxicol. 2018 Mar 16;19:10. doi: 10.1186/s40360-018-0200-y (PMC5857122; doi:10.1186/s40360-018-0200-y)
Supplement: Supplementary file 2 — Table S1. Schedule of study assessments. (PDF 69 kb) [file 40360_2018_200_MOESM2_ESM.pdf]

Table S1. Schedule of study assessments

		Confinement						
**Visit**	**Screen**	**Check-in**	**Lead-in**		**Treatment period**	**Washout**	**Discharge**	**Follow-up***
Study day	**−28 to −04**	**−03**	**−02**	**−01**	**1 to 12**	**13, 14**	**15**	
Informed consent	X							
Inclusion and exclusion criteria	X	X						
Demography and medical/medication history	X							
Physical examination	X	X					X[†]	
Randomisation					X			
Vital signs[‡]	X	X			X	X	X[†]	
Height and weight	X						X[†,¶]	
ECG (12-lead, in triplicate)	X	X			X	X	X[†]	
Biochemistry and haematology[§]	X	X			X	X	X[†]	
Urinalysis	X	X			X	X	X[†]	
Lipid panel	X	X			X	X	X[†]	
Coagulation	X	X			X	X	X[†]	
Thyroid panel	X	X				X	X[†]	
Vitamin A, D and E sampling**	X	X				X	X[†]	
HIV Ab, HBsAg and HCV Ab	X							
Pregnancy test (female participants)	X	X					X[†]	
Follicle-stimulating hormone	X							
Low-fibre, medium-fat diet			X	X	X	X		
Urine drug and alcohol screening	X[††]	X						
Study drug administration					X			
Pharmacokinetic blood sampling					X	X		
Bisacodyl administration[‡‡]				X		X		
Stool collection			X	X	X	X		
C4 sampling				X	X	X	X[†]	
Bristol Stool Chart assessment			X	X	X	X		
AEs/SAEs	X	X	X	X	X	X	X	X[‡]
Concomitant medication	X	X	X	X	X	X	X	X[‡]

*A follow-up telephone call was conducted 7 ± 2 days after the last dose of study drug.

[†]In the event that a participant prematurely discontinued participation in the study, every attempt was made to complete these assessments.

[‡]Vital signs included orthostatic measurements with a minimum of 3 min standing. Vital signs were assessed six times on days 1–15. At screening and on day −3, only single orthostatic measurements were taken.

[¶]Weight only.

[§]All biochemistry safety laboratory tests were to include glucose testing (after fasting from pre-dose on day 1 onward).

**etinol (vitamin A), 25-hydroxycholecalciferol (vitamin D) and α-tocopherol (vitamin E).

[††]Drugs of abuse were assessed at screening; on day −3 check-in to the clinical research centre, drugs of abuse were assessed and an alcohol breath test was performed.

[‡‡]A bisacodyl suppository was to be administered between 14:00 and 18:00 h on day −1 and day 14 if a stool sample had not been provided in the preceding 38 h.

Ab, antibodies; AE, adverse event; C4, 7α-hydroxy-cholesten-3-one; ECG, electrocardiography; HBsAg, hepatitis B surface antigen; HCV, hepatitis C virus; HIV, human immunodeficiency virus; SAE, serious adverse event.
