# Supplementary material for: A randomised, double-blind, placebo-controlled phase 1 study of the safety, tolerability and pharmacodynamics of volixibat in overweight and obese but otherwise healthy adults: implications for treatment of non-alcoholic steatohepatitis
Source: BMC Pharmacol Toxicol. 2018 Mar 16;19:10. doi: 10.1186/s40360-018-0200-y (PMC5857122; doi:10.1186/s40360-018-0200-y)
Supplement: Supplementary file 3 — Figure S1. Frequency of stool hardness during treatment (Bristol Stool Chart scores). (PDF 1081 kb) [file 40360_2018_200_MOESM3_ESM.pdf]

Figure S1. Frequency of stool hardness during treatment (Bristol Stool Chart scores)

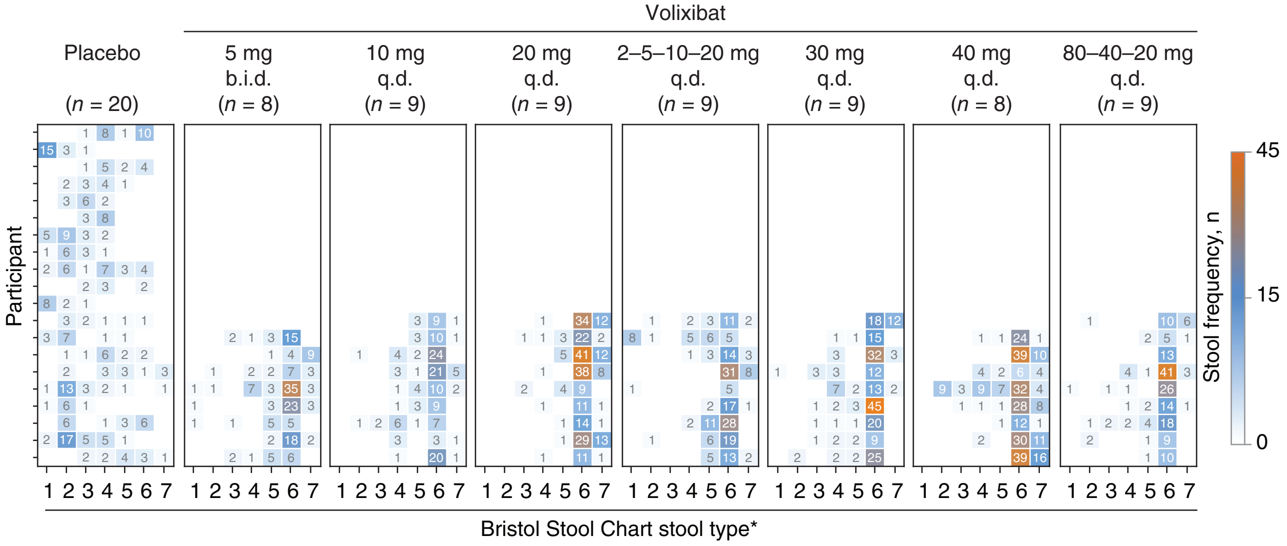

The number (n) in each shaded box and the colour of each box denote stool frequency according to the colour scale on the right; each participant is unique in each of the treatment groups.

*Type 1 is the hardest stool type and type 7 is the softest stool type.

b.i.d., twice daily; q.d., once daily
